# Microwave-induced resistance oscillations and zero resistance states in 2D bilayer systems

**DOI:** 10.1186/1556-276X-8-259

**Published:** 2013-05-29

**Authors:** Jesus Iñarrea

**Affiliations:** 1 Escuela Politécnica Superior, Universidad Carlos III

**Keywords:** Bilayer systems, Microwaves, Magnetoresistance

## Abstract

We report on theoretical studies of recently obtained experimental results on microwave-induced resistance oscillations and zero resistance states in Hall bars with two occupied subbands. In these experiments, resistance presents a peculiar shape which appears to have a built-in interference effect not observed before. We apply the microwave-driven electron orbit model, which implies a radiation-driven oscillation of the two-dimensional electron system. Thus, we calculate different intra- and inter-subband electron scattering rates revealing different microwave-driven oscillations frequencies for the two electronic subbands. Calculated results are in good agreement with experiments.

## Background

In the last decade, it was discovered that when a Hall bar (a 2DES with a uniform and perpendicular magnetic field (*B*) is irradiated with microwaves, some unexpected effects are revealed, deserving special attention from the condensed matter community: microwave-induced (MW) resistance oscillations (MIRO) and zero resistance states (ZRS) [1,2]. Different theories have been proposed to explain these striking effects [3-9] but the physical origin is still being questioned. On the other hand, a great effort has been made, specially from the experimental side, growing better samples, adding new features and different probes to the basic experimental setup, etc. [10-16]. One of the most interesting setups, carried out recently, consists in using samples with two or three occupied subbands [15]. These samples are either based in a double-quantum-well structure or just one single but wide quantum well.

The main difference in the longitudinal magnetoresistance (*R*_*xx*_) of a two-subband sample is the presence of magneto-intersubband oscillations (MISO) [17,18]. These oscillations occur due to periodic modulation of the probability of transitions through elastic scattering between Landau levels of different subbands [19-22]. Under MW irradiation, the first experimental results [16] of *R*_*xx*_ showed the interference of MISO and MIRO without reaching the ZRS regime. Later on, further experiments realized at higher MW intensities and mobility samples showed that the MW-response evolves into zero-resistance states for the first time in a two-occupied subband sample [15]. In the same experiment [15], it was also observed that there is a peculiar *R*_*xx*_ profile with different features regarding the one-subband case [1,2] affecting only valleys and peaks of MIRO’s in a surprising regular way, deserving special attention.

In this letter, we theoretically study magnetoresistance of a Hall bar being illuminated with MW radiation when two electronic subbands participate in the transport. We apply the theory developed by the authors, the *MW-driven electron orbits model*[3,23,24], which we extend to a two-subband scenario. According to this theory [3], when a Hall bar is illuminated, the electron orbit centers of the Landau states perform a classical trajectory consisting in a harmonic motion along the direction of the current (see ref. [3] for a detailed explanation).

In a double subband scenario, the situation gets more complicated but with a richer physics. On the one hand, due to the presence of MW, we have two 2DES (two subbands) moving harmonically at the MW frequency. On the other hand, we have two possible scattering processes with charged impurities: intra- and inter-subbands.

The competition between intra- and inter-subband scattering events under the presence of radiation alters significantly the transport properties of the sample. This is reflected in the *R*_*xx*_ profile through a strong and peculiar interference effect. As in experiments, our calculated results recover the presence of new features regularly spaced through the whole MIRO’s profile, mainly two shoulders at minima and narrower peaks.

## Methods

The MW driven electron orbits model, was developed to explain the *R*_*xx*_ response of an irradiated 2DEG at low *B*. We first obtained the exact solution of the corresponding electronic wave function [3,23-29]:

$\Psi_{N}(x,t)\propto \phi_{n}(x-X-x_{\text{cl}}(t),t)$, where *ϕ*_*n*_ is the solution for the Schrödinger equation of the unforced quantum harmonic oscillator, *X* is the center of the electron orbit. *x*_*cl*_(*t*) is the classical solution of a forced and damped harmonic oscillator [3-6,23,24]; $x_{\text{cl}}=\frac{eE_{0}}{m^{*}\sqrt{{\left(w_{c}^{2}-w^{2}\right)}^{2}+\gamma^{4}}}\cos\text{wt}=A\cos\text{wt}$, where *γ* is a phenomenologically-introduced damping factor for the electronic interaction with acoustic phonons, *E*_0_ is the amplitude of the MW-electric field, and *w* is the frequency of MW.

Thus, the electron orbit centers are not fixed, but they oscillate harmonically at *w*. This *radiation*−*driven* behavior will dramatically affect the charged impurity scattering and eventually the conductivity. Thus, we introduce the scattering suffered by the electrons due to charged impurities. If the scattering is weak, we can apply a time-dependent first-order perturbation theory. First, we calculate the impurity scattering rate [3-6,23,30] between two *oscillating* Landau states *Ψ*_*N*_ and *Ψ*_*M*_ belonging to the same subband, i.e., the intra-subband scattering rate $W_{n,m}^{\text{intra}}$ and to different subband, i.e., the inter- subband $W_{n,m}^{\text{inter}}$:

(1) $$\begin{array}{l} \;W_{n,m}^{\text{intra}}=|F_{\text{intra}}{|}^{2}\frac{e^{5}N_{i}Bm^{*}}{\hbar^{2}\varepsilon^{2}q_{0}^{2}}\left[1+2\overset{\infty }{\underset{s=1}{\sum }}e^{\left(\frac{-\mathrm{s\pi \Gamma}}{\hbar w_{c}}\right)}\right], \end{array}$$

(2) $$\begin{array}{l} \;W_{n,m}^{\text{inter}}=|F_{\text{inter}}{|}^{2}\frac{e^{5}N_{i}Bm^{*}}{\hbar^{2}\varepsilon^{2}q_{0}^{2}}\\ \;\times \left[1+2\overset{\infty }{\underset{s=1}{\sum }}e^{\left(\frac{-\mathrm{s\pi \Gamma}}{\hbar w_{c}}\right)}\cos\left(\frac{s2\pi \Delta_{12}}{\hbar w_{c}}\right)\right], \end{array}$$

*ε* being the dielectric constant, *N*_*i*_ the number of impurities, *Γ* the width of the Landau states, *Δ*_12_ the subband separation, and *q*_0_ as the Thomas-Fermi screening constant [31]. *F*_intra_ and *F*_inter_ are the form factors given by:

(3) $$\begin{array}{lll} F_{\text{intra}(\text{inter})} & =\int_{0}^{\infty }e^{-q(z-z_{i})}\Psi_{S}^{*}\Psi_{S(A)}\text{dz}\; & \;\\ & =\frac{e^{-\text{qd}}}{2}\left[{\left(\frac{b}{b+q}\right)}^{3}+(-){\left(\frac{b}{b-q}\right)}^{3}\right].\; \end{array}$$

To obtain the form factor expressions, we have considered at each side of the wide quantum well a triangular shape potential. Thus, we have applied the Fang-Howard approach (see ref. [31]) for the electronic wave function, where *b* is a variational parameter, and *q* is the electron wave vector exchanged in the scattering. *Ψ*_*S*(*A*)_ are the corresponding symmetric (antisymmetric) wave function of the wide quantum well. We have supposed a symmetrical delta doping, *d* being the average separation between the impurities and the 2DES at each side of the wide quantum well. With the experimental parameters at hand [15] and following the variational approach [31], we have carried out the calculation of the relative values of *F*_intra_ and *F*_inter_ resulting in |*F*_intra_|^2^≃3×|*F*_inter_|^2^. Next, we find the average effective distance advanced by the electron in every scattering jump, *ΔX*^*MW*^.

## Results and discussion

If we consider that the oscillation is at its mid-point when the electron jumps from the initial state and that it takes an average time $\langle \tau_{\text{intra}(\text{inter})}\rangle =\langle 1/W_{n,m}^{\text{intra}(\text{inter})}\rangle$ to get to the final one, then we can write for the average coordinate change in the *x* direction: $\Delta X_{\text{intra}(\text{inter})}^{\text{MW}}=\Delta X^{0}+A\cos(w\langle \tau_{\text{intra}(\text{inter})}\rangle )$, where *ΔX*^0^ is the effective distance advanced when there is no MW field present.

Then, we calculate average values of the intra and inter-subband scatering rates and obtain a direct relationship given by $\langle W_{n,m}^{\text{intra}}\rangle \approx 3\times \langle W_{n,m}^{\text{inter}}\rangle \Rightarrow \langle \tau_{\text{intra}}\rangle \approx \frac{1}{3}\langle \tau_{\text{inter}}\rangle$, where we have considered that the cosine average value, $\langle \cos\frac{s2\pi \Delta_{12}}{\hbar w_{c}}\rangle \to 0$ for $\Delta_{12}>\hbar w_{c}$, and we have carried out the sum $\overset{\infty }{\underset{s=1}{\sum }}e^{\left(\frac{-\mathrm{s\pi \Gamma}}{\hbar w_{c}}\right)}\to \frac{\text{exp}(\frac{-\mathrm{s\pi \Gamma}}{\hbar w_{c}})}{1-\text{exp}(\frac{-\mathrm{s\pi \Gamma}}{\hbar w_{c}})}$. We have taken an average value for the variational parameter $\bar{b}=0.3$ nm ^−1^, meaning an average width for the two lateral triangular shape wells of 〈*z*〉=10–12 nm [31].

(4) $$\begin{array}{lll} \;\sigma_{\text{xx}} & =\frac{6e^{7}m^{*2}BN_{i}}{\pi \varepsilon^{2}\hbar^{6}q_{0}}{\left[\Delta X^{0}+A\cos\frac{1}{3}w\langle \tau_{\text{inter}}\rangle \right]}^{2}\; & \;\\ & \;\;\;\;\times \left[\right)1+2e^{\frac{-\pi \Gamma }{\hbar w_{c}}}+e^{\frac{-\pi \Gamma }{\hbar w_{c}}}\frac{X_{S}}{\sinh X_{S}}\; & \;\\ & \;\;\;\;\left(\cos\frac{2\pi (E_{F}-E_{1})}{\hbar w_{c}}+\cos\frac{2\pi (E_{F}-E_{2})}{\hbar w_{c}}\right)])\; & \;\\ & \;\;\;\;+\frac{2e^{7}m^{*2}BN_{i}}{\pi \varepsilon^{2}\hbar^{6}q_{0}}{\left[\Delta X^{0}+A\cos w\langle \tau_{\text{inter}}\rangle \right]}^{2}\; & \;\\ & \;\;\;\;\times \left[\right)1+2e^{\frac{-\pi \Gamma }{\hbar w_{c}}}\cos\frac{2\pi \Delta_{12}}{\hbar w_{c}}+e^{\frac{-\pi \Gamma }{\hbar w_{c}}}\frac{X_{S}}{\sinh X_{S}}\; & \;\\ & \;\;\;\;\left(\cos\frac{2\pi (E_{F}-E_{1})}{\hbar w_{c}}+\cos\frac{2\pi (E_{F}-E_{2})}{\hbar w_{c}}\right)]),\; \end{array}$$

where $X_{S}=\frac{2\pi^{2}k_{B}T}{\hbar w_{c}}$ and *E*_1_ and *E*_2_ are the energies of the first and the second subbands, respectively. This equation shows the physical equivalence to a situation with only one scattering time and two different oscillations frequencies for the MW-driven subbands: *w*/3 for the intra-subband and *w* for the inter-subband scattering rate [32,33]. They demonstrate also the origin for the regular and strong interference profile observed in experiments where the factor 1/3 is essential to obtain the interference effect regularly spaced affecting only valleys and peaks. A different factor would produce a totally distinct interference and also distinct *R*_*xx*_ response. This factor comes from the calculation of the squared magnitude of the corresponding form factors which eventually determine the different scattering rates between the intra-subband and the inter-subband processes.

In physical terms,during the scattering jump, the electron *perceives* approximately three times faster MW-driven oscillation of the 2DES when is inter-subband with respect to the intra-subband. Then, we are going to obtain a MIRO profile made up of two different MW frequencies, as if the sample was illuminated by two different radiation sources at the same time. This gives rise to a clear interference effect reflected in the final *R*_*xx*_ profile. To obtain *R*_*xx*_, we use the relation $R_{\text{xx}}=\frac{\sigma_{\text{xx}}}{\sigma_{\text{xx}}^{2}+\sigma_{\text{xy}}^{2}}≃\frac{\sigma_{\text{xx}}}{\sigma_{\text{xy}}^{2}}$, where $\sigma_{\text{xy}}≃\frac{n_{i}e}{B}$ and *σ*_*xx*_≪*σ*_*xy*_.

**Figure 1:**
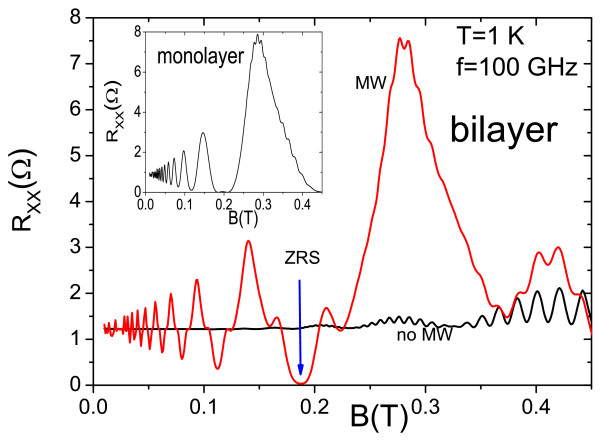
**Calculated *R***_***xx ***_**vs *B* for dark (no MW) and MW situations.** The ZRS is marked with an arrow. The MW frequency is 100 GHz. We observe clearly the peculiar features for bilayer systems: shoulders at minima and narrower peaks regarding the single occupied subband case (see inset). Shoulders and narrow peaks are the outcomes of the interference between the intra- and inter-subband scattering processes.

In Figure 1, we present calculated *R*_*xx*_ vs *B* for dark and MW situations and frequency *f*=*w*/2*π*=100 GHz. We can observe MISO peaks for the dark curve, MIRO for the MW curve, and the ZRS marked with an arrow. We observe the new features appearing regularly spaced in peaks and valleys for bilayer systems: two nearly symmetric shoulders in valleys and narrower peaks with respect to the single occupied subband case (see inset). According to our model, these new features are results of the interference between the competing intra- and inter-subband scattering processes. In valleys, we observe a constructive interference effect giving rise to two shoulders, meaning more current through the sample; meanwhile, the narrower peaks mean a destructive interference and less current.

## Conclusions

In summary, we have theoretically studied the recently discovered microwave-induced resistance oscillations and zero resistance states in Hall bars with bilayer systems. Resistance presents a peculiar shape which appears to have an interference effect not observed before. Applying the microwave-driven electron orbit model, we calculate different intra- and inter-subband electron scattering rates under MW, revealing that the former is three times greater than the latter. This is physically equivalent to different microwave-driven oscillation frequencies for the two electronic subbands.

## Competing interests

The author has no competing interests.
